# Adaptive Control of the Packet Transmission Period with Solar Energy Harvesting Prediction in Wireless Sensor Networks

**DOI:** 10.3390/s150509741

**Published:** 2015-04-24

**Authors:** Kideok Kwon, Jihoon Yang, Younghwan Yoo

**Affiliations:** Department of Computer Engineering, Pusan National University, Busandaehak-ro 63beon-gil Geumjeong-gu, Busan 609-735, Korea

**Keywords:** WSN, energy harvesting, solar energy, packet transmission period control

## Abstract

A number of research works has studied packet scheduling policies in energy scavenging wireless sensor networks, based on the predicted amount of harvested energy. Most of them aim to achieve energy neutrality, which means that an embedded system can operate perpetually while meeting application requirements. Unlike other renewable energy sources, solar energy has the feature of distinct periodicity in the amount of harvested energy over a day. Using this feature, this paper proposes a packet transmission control policy that can enhance the network performance while keeping sensor nodes alive. Furthermore, this paper suggests a novel solar energy prediction method that exploits the relation between cloudiness and solar radiation. The experimental results and analyses show that the proposed packet transmission policy outperforms others in terms of the deadline miss rate and data throughput. Furthermore, the proposed solar energy prediction method can predict more accurately than others by 6.92%.

## 1. Introduction

Energy management is one of the essential factors in an embedded system due to the energy limitation. In wireless sensor networks (WSNs), sensor nodes collect various environmental data, such as temperature, humidity, sound and pressure. The faster the sensed data are sent to the server, the more valuable the data are, as they reflect the current situation more accurately. However, the data may not be delivered always the minute that it is collected due to the energy constraint of sensor nodes. A WSN suffers from the problem of small device dimensions. The device dimensions are related to the energy management, since a large battery cannot be equipped. Although many researchers have studied how to improve energy efficiency and how to enhance battery capacity, the lifetime of sensor nodes is still limited. Thus, an alternative technology is garnering attention, which is an energy harvesting system that acquires energy from various environmental sources, like the Sun, wind or pressure [1]. The past energy management in WSN focused on saving energy to prevent battery discharge. On the other hand, in the energy harvesting system, it is important to keep an energy-neutral policy, which means that the sensor node permanently maintains the performance demanded by user applications without power dissipation [2].

For an effective energy neutral policy, the system should be able to accurately predict the amount of energy that will be harvested in the future. The prediction methods are categorized depending on the type of utilized information. Some research predicts the current energy harvesting efficiency based on the harvested energy in the past. The others use weather information. These kinds of prediction methods provide better performance than the former, since they consider weather factors that are changing quickly in a short time. This prediction, however, requires high computational complexity and needs to sense environmental data for a long time to find a particular coefficient, although it is accurate within a variety of times ranging from three hours to a week.

This paper consists of two parts: energy harvesting prediction and data transmission period control. First, we predict the amount of harvested energy for the next three hours by using simple weather information. While existing prediction methods require a lot of weather factors, our method leverages just a few weather factors, which are the information of the past insolation and the cloud amount. From the relation between the two kinds of information, the amount of future solar radiation is deduced, so the proposed prediction method need not have a high computational or sensing overhead despite its high accuracy.

Second, we propose ACSE (adaptive control of packet transmission period with solar energy harvesting prediction) to control the packet transmission period based on our energy harvesting prediction. The basic philosophy is very simple: when energy is harvested plentifully, sensor nodes increase the throughput by shortening the transmission period. In the opposite case, they reduce energy consumption by lengthening the transmission period. ACSE aims to maintain the performance permanently while enhancing network throughput in the case of abundant solar insolation. It is easier to estimate solar energy harvesting than other renewable energy, because it has obvious features, like the distinct periodicity over a day. Such a feature is used by sensors to get a balance between throughput and energy consumption. As a result, the sensor node can maximally utilize the battery, relieving the worry about their battery dissipation.

The rest of the paper is organized as follows: Section 2 examines the related work about energy harvesting prediction and the packet scheduling policy considering the amount of harvested energy. Section 3 proposes a solar energy prediction method based on cloudiness and analyzes its accuracy. Section 4 suggests our transmission period control policy based on solar energy prediction. In Section 5, the simulation result shows that our method has better energy efficiency and meets the deadline of real-time works relatively well. Finally, Section 6 concludes this paper.

## 2. Related Work

### 2.1. Solar Energy Harvesting Prediction Model

EWMA (energy prediction model using exponentially weighted moving-average) uses the profile about the amount of harvested energy in the past. The basic idea is that the amount of harvested energy does not change remarkably in a short time. EWMA splits a day into 48 sections from zero to 47, and each section is 30 min long. When *x_k_* denotes the amount of the energy harvested at the *k*-th section and *a* is a weighting factor, the average of the harvested energy at each section is as in Equation (1). $\bar{x_{k}}$ means the *k*-th variance of the estimated harvesting energy. Here, how to decide the weighting factor *a* is very important, because the prediction accuracy totally depends on it. However, EWMA cannot dynamically react to environmental change.

(1) $$\bar{x_{k}}=a\bar{x_{k-1}}+\left(1-a\right)x_{k}$$

The method called cloudy computing [3] predicts the amount of harvested solar energy, using weather information. It is shown that EWMA is not accurate enough for the recent future within 3 h. This method uses two main coefficients to predict harvested energy. One coefficient considers the location of the specific region, since the solar insolation is affected by the geographical location on the Earth. The other indicates how clear the sky is. It is insisted that the amount of harvested energy may be linearly proportional to the clearness. However, we will prove that this is not so simple through the thorough investigation of the past data.

Most WSN research focuses on reducing the energy consumption of sensors, but does not consider the future harvested energy. For instance, SEA-OR (Spectrum and Energy Aware Opportunistic Routing) [4] considers the physical location, the residual energy level and the link reliability to decide the back off-time of the sensor nodes, but future energy was not been taken into account. On the other hand, our method can effectively manage the energy in sensor batteries by considering the energy harvested in the near future, preventing the battery dissipation and harvested energy dumping due to the battery being fully charged.

### 2.2. WSN Policy Based on Energy Harvesting Prediction

Hsu *et al.* [5] suggests a power control policy in energy harvesting systems. For energy neutrality, they control the duty cycle of sensor nodes by making them switch between the active and the sleep mode repeatedly. However, they consider only constant data generation, so this is not suitable for real-time services, such as intrusion detection and volcano eruption monitoring.

LSA (lazy scheduling algorithm) [6] schedules packet transmission considering not only energy, but also the packet deadline. The authors insist on using lazy scheduling to solve the problem of the lack of energy, which can occur if greedy scheduling is applied to energy harvesting networks. However, the method may often miss the deadline of burst data.

The papers above have in common the assumption of constant data generation only. This assumption does not match real-time applications in WSN. Furthermore, they assume that the amount of the harvested energy can be predicted very accurately. However, this is dangerous, since solar energy significantly fluctuates depending on the weather. Our study relieves those assumptions and overcomes such limitations.

## 3. Energy Harvesting Prediction

**Figure 1:**
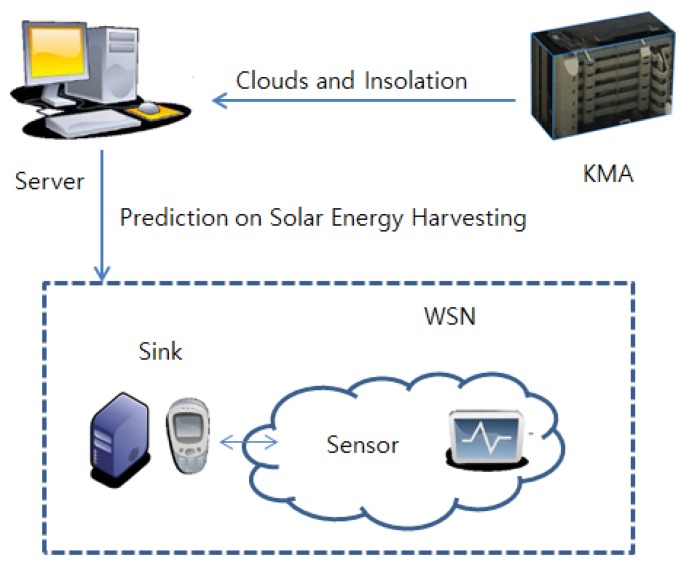
The system overview.

The proposed scheme predicts the future insolation by analyzing the relation between the past insolation and the cloud amount. Cloudy computing [3] has to collect the sunshine intensity at a specific area for several months to predict harvested energy. On the contrary, our method brings past weather information from KMA (Korea Meteorological Administration), as shown in Figure 1, without collecting this large amount of data. Thus, this system can be quickly deployed and used in any region without much advance preparation. Furthermore, the server estimates the future insolation based on the KMA data on behalf of sensor nodes and broadcasts it to the sensors. Therefore, each sensor does not have to receive a large amount of data from KMA and can save its energy for computation. In addition, since the server was designed to deal with weather data presented in the XML format (KMA provides the data in XML format), our system can be used in any country if their meteorological administration provides the weather data in an XML-compatible format. However, there may be an issue regarding the accuracy of the KMA data, since KMA provides data, not for the small region covered by a WSN, but for a large area, like a city. Moreover, the KMA data are sensed every hour on the hour, not at every instant. Currently, we argue that this kind of inaccuracy is a trade-off for the simple architecture and easy usage of the proposed system. Later, if the data for the difference between KMA information and sensed values are accumulated for a long time, the inaccuracy can be overcome by adopting a linear regression method.

**Figure 2:**
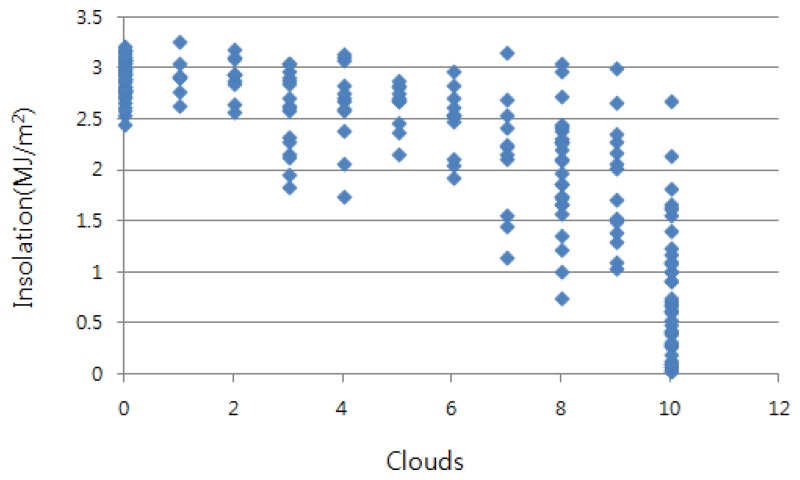
The insolation depending on the cloud amount in June from 2003 to 2012.

**Figure 3:**
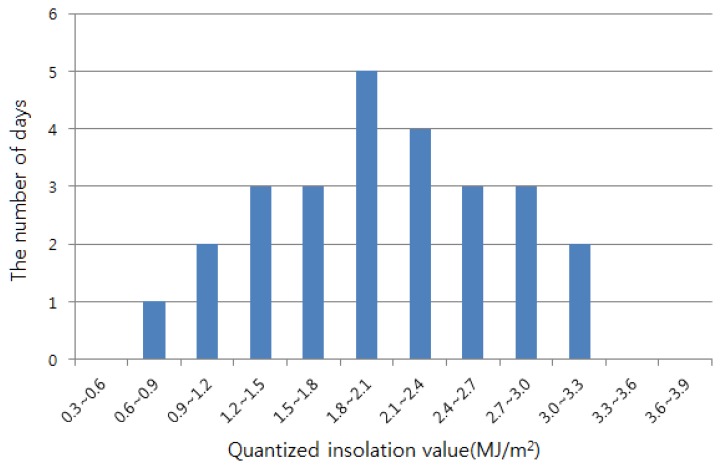
Quantized insolation distribution.

KMA measures the cloud amount and insolation every hour. Furthermore, they provide their own prediction on cloud amount and insolation for the next three hours. The amount of cloud is scaled from zero to 10. The closer to 10 it is, the more clouds there are. Figure 2 shows the distribution of pairs of cloudiness and insolation in June from 2003 to 2012. We can see that the insolation is tightly related to the amount of cloud. The more cloud there is, the lower the insolation intensity is. Our method predicts the insolation intensity by using this relationship. This procedure consists of four steps. Let us examine each step with an example:

1. Decide the date and time we want to predict the solar insolation.
  The time range for which we can predict insolation is limited to three hours in the future, because we can acquire only three-hour prediction on cloudiness from KMA. Let us assume that we want to predict the insolation in Busan, Korea, at 3 p.m., 2 August 2013.
2. Get the past data on insolation and cloudiness from KMA.
  The insolation intensity is largely affected by the Sun's altitude, and the altitude changes with the Earth's revolution around the Sun. Thus, we get the data of 20 days around the targeting date or, in the previous example, the data of 10 days before and after 2 August (from 23 July to 12 August) are used to predict the future insolation. We also need to decide how many years' data will be used; then, we found out, through our experiment, that the prediction accuracy is not improved linearly in proportion to the amount of data. Therefore, we decide to get the data of the recent 10 years, as the prediction accuracy is not noticeably improved although more than 10 years' data are used.
3. Compute the average of insolation over the days that were in a similar situation in the past.
  As mentioned above, we got a total of 200 data (10 years × 20 days/year) from the KMA database, but we do not use all of the data. First, we filter out the days for which the cloudiness situation was different from the target instant. If the degree of cloudiness was predicted as seven by KMA at the aforementioned instant, 3 p.m., 2 August 2013, we extract only the data of the days whose cloudiness was seven. Figure 3 shows this screening result, with the number of days being counted for each quantized insolation intensity value (MJ/m^2^). For instance, the number of days for which the insolation value was between 1.8 and 2.1 is five. Eventually, we can estimate the insolation at the target instant by averaging all of the insolation intensity values in Figure 3.
4. Complement the estimated insolation according to the required confidence level.
  In the last step, the average of the previous insolation intensities was adopted as the predicted insolation at the target instant. However, it is a little dangerous to use this value, as it is in the real-time embedded system, because the real insolation can be weaker than the estimated value. In this case, the system consumes more energy than the harvested one, resulting in the death of the real-time system. Therefore, we need to prevent the dissipation of the entire amount of energy by adopting a small conservative value as the estimated insolation. We calculated the confidence intervals of the estimated insolation with various confidence levels, *α* = 0.7, 0.8 and 0.9, as follows:
  (2) $$\begin{array}{cc} X∼N\left(\mu ,\sigma^{2}\right), & f\left(x\right)=\frac{1}{\sigma \sqrt{2\pi }}e^{-{\left(x-\mu \right)}^{2}/\sigma^{2}} \end{array}$$
  (3) maxa,P(X≥a)≥α

**Figure 4:**
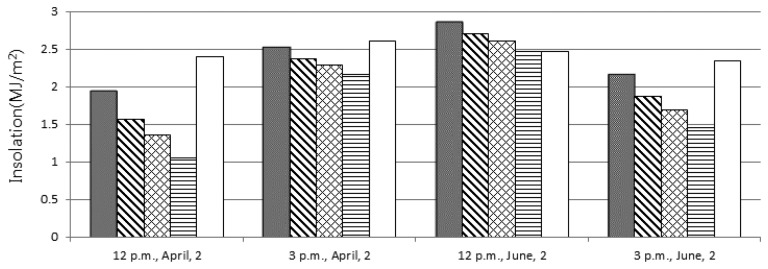
The comparison with the lower bounds of confidence intervals.

Figure 4 compares the actual insolation with the original estimated value and the lower bound of each confidence interval. The actual values are always greater than the lower bounds with the confidence level = 0.9, while they go beyond the confidence intervals associated with the other confidence levels sometimes. As a result, it will be safe to adopt the lower bound of the confidence level = 0.9 as the estimated value. However, note that too conservative policies yield low network throughput by forcing the system to save too much energy even when enough residual energy remains in the battery.

**Figure 5:**
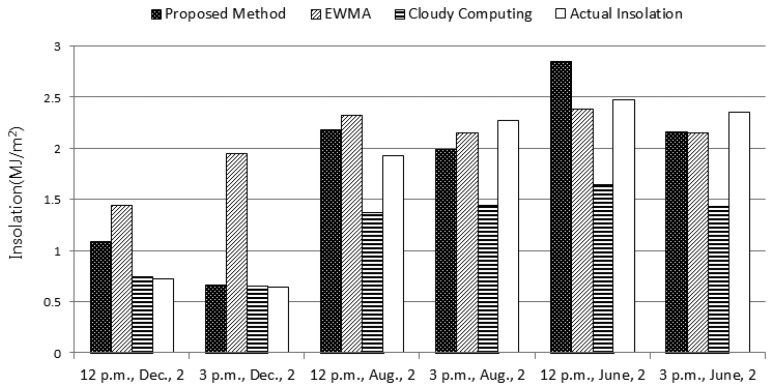
The comparison with existing algorithms.

Figure 5 illustrates the insolation estimated by several schemes. For the proposed method, the original estimation values without the complementation using any confidence level are used. Although our method overestimates the insolation sometimes, the accuracy is very high in comparison with others. The accuracy of the proposed method and cloudy computing are 85.02% and 78.09%, respectively, the difference being 6.92%. Particularly, EWMA excessively underestimates the insolation, with only an accuracy of 40.52%, which may degrade the performance of the WSN too much.

## 4. ACSE: Packet Transmission Period Control

This section suggests how to control the packet transmission period based on the prediction result in Section 3. Note that our transmission period control method, called ACSE, does not have to be combined with our own energy harvesting prediction all of the time. Provided that another prediction method is more accurate than our method, ACSE may give better performance by adopting it.

### 4.1. WSN and Task Model

**Figure 6:**
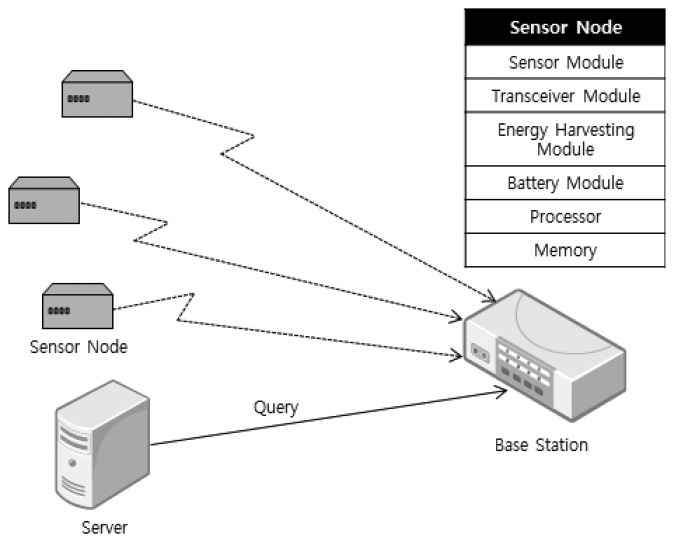
Energy harvesting sensor network model.

Our model is similar to [7] in which a base station exists, and each sensor node has a periodic and an aperiodic task. Figure 6 shows a system consisting of a sensor, a transceiver, an energy harvesting module, a battery, a processor and memory. Each node sends packets to the BS (base station), and then, the BS forwards them to the server after data aggregation. The energy level in the energy storage module is between *B^min^* and *B^max^*. *H*(*t*, *h*) in Equation (4) denotes the estimated energy to be harvested for *h* hours from *t*, and *p*(*t*) is a function to predict the amount of harvested energy for one hour starting from time *t*.

(4) $$\begin{array}{lll} H\left(t,h\right) & =\int_{t}^{t+h}p\left(x\right)dx, & p\left(t\right):a\text{prediction}\;\text{function}\\ & =\overset{t+1+h}{\underset{x=t+1}{\text{∑}}}p\left(x\right) & \end{array}$$

- An aperiodic task: A sensor node generates packets either when the condition is met or when it receives a server query. An example of the aperiodic task is: “If the temperature is over 30 °C, report its temperature immediately”. When *E* and *R* denote the packet transmission energy and the average number of aperiodic events per hour, the total energy for *h* hours is defined as:
  (5) C(h)=E×R×h
- A periodic task: This task is to send a packet to the BS periodically. An example is: “Report the temperature every 30 min for the next 5 h”. Each periodic task *x* is numbered from one to *n*. *T_x_* denotes the transmission period of the periodic task *x*. Each task has a transmission deadline; thus, it has the minimum and the maximum period, $T_{x}^{\text{min}}$ and $T_{x}^{\text{max}}$. For the periodic task, the total energy consumption for *h* hours is computed as:
  (6) $$\begin{array}{cc} D\left(h\right)=hE\times \underset{x}{\text{∑}}\frac{1}{T_{x}}, & T_{x}^{\text{min}} \end{array}\leq T_{x}\leq T_{x}^{\text{max}}$$

### 4.2. Adaptive Transmission Period Control

**Figure 7:**
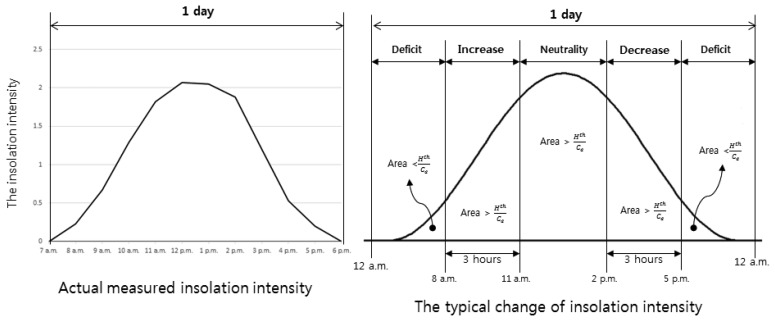
The insolation intensity for a day.

The insolation intensity for a day typically has the form of a bell curve, like Figure 7, unless the weather changes abruptly during the daytime. The actual harvested energy is proportional to both the intensity and the conversion efficiency of the solar module. In the figure, *H^th^* and *c_e_* denote the energy that the sensor application requires and the convergence efficiency of the solar module.

We divided the energy harvesting situations into four types: increase, decrease, neutrality and deficit. In the first three types, more energy is harvested than that required by the applications. They were subdivided into three types depending on the estimated change of insolation for the next three hours. If the insolation were to likely increase continuously, it is the increase type. The opposite case is the decrease type. If the insolation were guessed to fluctuate, it is the neutrality type. On the other hand, in the deficit type, more energy is used than the harvested energy.

**Figure 8:**
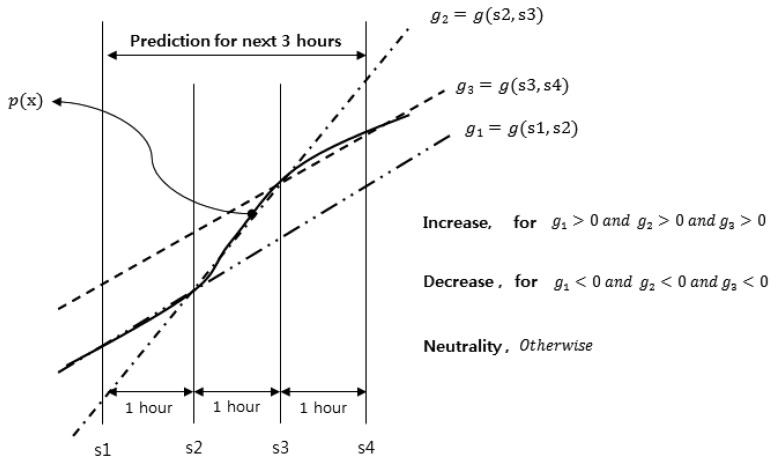
Decision for three energy harvesting types: increase, decrease and neutrality.

Figure 8 depicts in detail how the energy harvesting type can be determined for the cases where the harvested energy is larger than the needed energy. This case includes the increase, decrease and neutrality types. For this decision, the gradient of the insolation change is calculated like this:

(7) $$g\left(x_{1},x_{2}\right)=\frac{p\left(x_{2}\right)-p\left(x_{1}\right)}{x_{2}-x_{1}}$$

where *p*(*x_k_*) is the aforementioned prediction function on the insolation at *x_k_*. The insolation is predicted every hour.

- Increase type: The amount of harvested energy is expected to continuously increase for the next three hours. Generally, it happens in the morning.
- Decrease type: Oppositely, as observed in the afternoon, the harvested energy is expected to continuously decrease for the next three hours
- Neutrality type: The harvested energy dose not continuously increase nor decrease for the next three hours. Namely, the harvested energy is predicted to fluctuate for the next three hours.

As mentioned earlier, if less energy is harvested than the energy needed, it is classified as the deficit type.

Our method, ACSE, dynamically controls the packet transmission of periodic tasks, depending on the energy harvesting types. The goal is to keep a WSN alive even when solar energy is not harvested at all, so that important aperiodic packets may be delivered in real time. At the same time, the throughput of periodic tasks should be maximized. Thus, ACSE adopts a different scheduling policy depending on the energy harvesting type. The scheduling policies will be described in detail later. Before that, the notations, listed in Table 1, are introduced.

(8) $$A\left(h\right)=H\left(t^{\text{cur}},h\right)$$

(9) $$B^{p}\left(h\right)=B^{\text{cur}}-B^{\text{min}}+A\left(h\right)-C^{\text{max}}\left(h\right)$$

The energy increasing and decreasing ratio can be figured out by using the equations in Equations (8)∼(10), where *A*(*h*) and *B^p^*(*h*) denote the total harvested energy and the maximum energy that the periodic tasks can use for the next *h* hours. *B^cur^* is the current residual energy, and *B^min^* is the minimum energy for a sensor node to keep running without harvested energy for some duration. In our method, this *B^min^* was defined as the energy that the periodic tasks can use for one month with the longest possible period of packet generation, because the rainy season in Korea is rarely longer than one month. Thus, the minimum energy guarantees that a sensor node can provide its minimum service even if it cannot harvest energy at all for one month. Then, *C^max^*(*h*) denotes the maximum energy that aperiodic tasks may use for the next *h* hours. Actually, it is impossible to know how many aperiodic tasks will happen in advance, so we assume that they follow the Poisson distribution. Given an arrival rate λ, we compute the maximum number of events that can happen for the next *h* hours with a higher chance above 95%. Our method will be evaluated in the next section with various arrival rates.

*B^p^*(*h*) is the energy that the periodic tasks can use for the next *h* hours. It is compared with *D*(*h*), which is the energy that the periodic tasks will use for the next *h* hours assuming that they maintain the current period. If *B^p^*(*h*) is less than *D*(*h*), then a sensor node needs to use the longer period of its periodic task to prevent entire energy dissipation. Otherwise, the period can be shortened to increase the throughput, because the battery energy is enough. The ratio between *B^p^*(*h*) and *D*(*h*) is defined as the proportional factor *α* in Equation (10). The energy consumption for the next *h* hours is increased or decreased depending on the value of *α*. If *α* is greater than one, the period of periodic tasks is shortened, because more available energy is expected for the next *h* hours.

(10) $$\alpha =\frac{B^{p}\left(h\right)}{D\left(h\right)}$$

ACSE has four period control policies corresponding to the four different solar energy harvesting types.

- Neutrality policy: This policy is adopted in the neutrality type. Although the insolation intensity is not stable, the harvested energy is expected to be larger than or equal to the requested energy. Thus, the current periods are maintained for the next *h* hours.
- Increase policy: More and more energy is expected to be harvested continuously for the next *h* hours. Thus, each periodic task can transmit *α* times as many packets as the current number of packets. In Equation (11), *T_x_* and $\bar{T_{x}}$ denote the current period and the new period, and *n* is the number of periodic task applications in the sensor node. The *α* is greater than one.
  (11) $$\begin{array}{ccc} \bar{T_{x}}=\frac{1}{\alpha }\times T_{x}, & T_{x}^{\text{min}}\leq \bar{T_{x}}\leq T_{x}^{\text{max}}, & \text{for}\;x=1,\cdots ,n \end{array}$$
- Decrease policy: Although the harvested energy is still greater than the needed energy, the insolation intensity is continuously weakened. Thus, the battery can be totally dissipated if the current period is kept for a long time. The periodic tasks lengthen their periods by using *α* smaller than one in Equation (11).
Deficit policy: In the deficit type, less energy is harvested than the required energy, so staying alive until the deficit type ends is the goal. Instead of predicting for the next *h* hours, simply, ACSE firstly estimates *h^r^*, which is the remaining time until the solar energy harvesting type will be changed into other types than the deficit type, by leveraging the weather information. Then, the proportional factor *α* that is computed between *B^p^*(*h^r^*) and *D*(*h^r^*) is used to control the packet transmission period in Equation (11). Note that *B^p^*(*h^r^*) and *D*(*h^r^*) denote the total energy that the periodic tasks can use and the energy required to keep the current period until enough energy can be harvested again.

(12) $$\begin{array}{ll} B^{p}\left(h^{r}\right) & =B^{cur}-B^{\text{min}}+A\left(h^{r}\right)-C^{\text{max}}\left(h^{r}\right)\\ \alpha & =\frac{B^{p}\left(h^{r}\right)}{D\left(h^{r}\right)} \end{array}$$

## 5. Performance Evaluation

Using MATLAB, we performed the simulation to evaluate ACSE in terms of energy efficiency and throughput. Therefore, before that, we needed to estimate the amount of energy that might be harvested during the simulation time. Setting the simulation to continue for five days from 8 a.m., 4 May 2014, we compared our estimated insolation intensity with the KMA's report. In short, our scheme in Section 3 could predict the future insolation fairly well with a difference of only 6.78% from the actual value. Each sensor node had three periodic tasks and an aperiodic task that generated packets following the Poisson distribution. Other experimental parameters are given in Table 2.

The compared methods are the greedy method, lazy method and optimal packet scheduling [8]. The greedy method focuses on the transmission throughput without considering residual energy. It tries to maximize the throughput as long as the battery power is available. On the contrary, the lazy method emphasizes the long lifetime of the system rather than the network throughput, delaying packet transmission as much as possible. Lastly, the optimal scheduling adjusts transmission periods so that the total transmission time of all packets may be minimized, based on accurate energy harvesting information. However, they did not propose any energy prediction method. In addition, they need to know entire packets that they will send in advance; thus, they cannot handle aperiodic packets generated irregularly.

**Figure 9:**
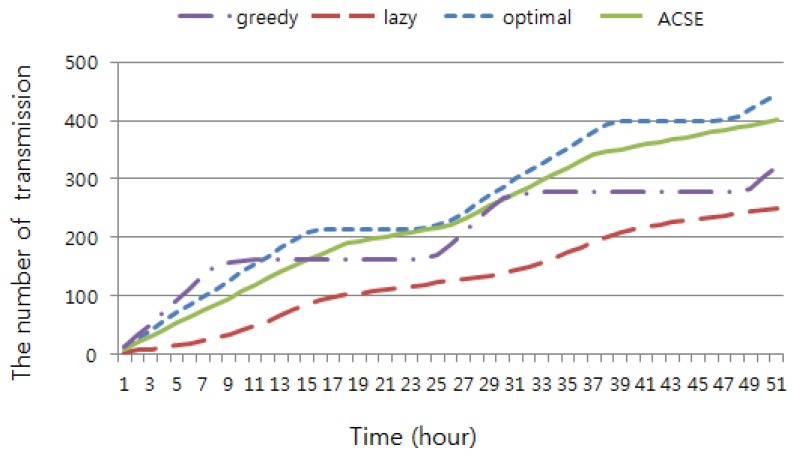
The number of packet transmissions.

Figure 9 shows the variance of transmission numbers against the simulation time. At first, the greedy method sent more packets than the others, but the number of transmissions became stable during some intervals, e.g., 9 to 25 and 31 to 47 h from the starting time. The greedy method consumed all of the energy very quickly, and it could not send more packets until the battery was recharged. Solar energy cannot be harvested at night. Furthermore, the solar radiation in the early morning and in the late afternoon was not strong enough to harvest as much as the energy needed for packet transmission. On the other hand, the lazy method held off packet transmissions throughout the simulation so long as their deadlines had not passed. Conclusively, as time grew, the optimal and the proposed ACSE showed the best performance in terms of throughput. Note that, mentioned before, the optimal method needs several strict assumptions, e.g., entire packet generation information and accurate estimation of harvested energy.

**Figure 10:**
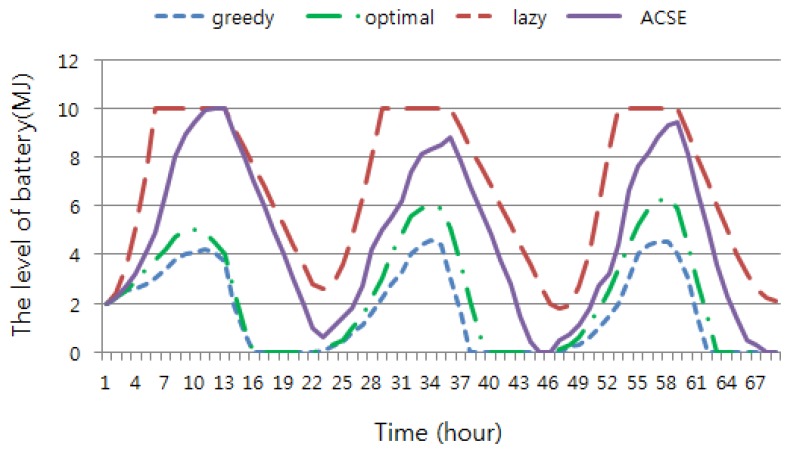
The change of the battery level.

Figure 10 illustrates the trace of the battery energy level change. The greedy method stayed at the complete discharge of the battery for a long time. The optimal method also often made the battery dissipate completely, since it is based on the assumption that the harvested energy can be accurately estimated. Our experiment showed an error of 6.78% compared with the actual harvested energy. On the other hand, the lazy method stayed at the full-charge of battery for a long time at the cost of low throughput. In this state, more solar energy is overflowed, which may have been stored and used by other methods. Unlike all other methods, ACSE always maintained the battery state between full charge and complete discharge. Thus, most aperiodic packets were successfully delivered even in the nighttime.

We observed the number of packets that missed the deadline due to the lack of energy. This experiment continued for five days and was repeated 30 times. As mentioned earlier, each sensor ran three periodic tasks and aperiodic tasks generated following the Poisson distribution. The arrival rates, λ, were set to 4, 8 and 12, and the results are given in Table 3. When λ = 4, ACSE missed the deadline of only three packets among a total of 491 packets, the success ratio being 99.4%. The greedy and optimal method could not satisfy the deadlines of packets frequently due to the battery dissipation, as shown in Figure 10. On the other hand, the lazy method satisfies the deadline best at the cost of throughput, since it pursues only the saving of energy for unexpected future use.

Besides the similar performance to the optimal scheduling in terms of throughput, ACSE also has a lower deadline miss ratio. These advantages are because ACSE dynamically controls transmission periods considering not only residual energy, but also the variance of harvested energy. Therefore, we argue that the proposed method ACSE can provide reasonable and realistic performance for energy harvesting wireless sensor network applications.

## 6. Conclusions

Our study suggests a novel estimation scheme for energy harvesting within three hours and ACSE for controlling packet transmissions according to the estimated energy. The estimation scheme utilizes weather factors, such as the past insolation and cloudiness. The scheme analyzes the correlation between these factors with consideration of the Earth's rotation. On the other hand, ACSE controls the packet transmission adaptively based on the estimated harvested energy. In our experiment, the integration of those schemes not only improved the prediction accuracy, but also enhanced the network throughput. Additionally, ACSE showed a lower deadline missing ratio and a higher energy utilization than the compared methods. Therefore, we can say that ACSE is an approach that can be used in a practical network. In the future, we have to handle two more issues: First, our prediction scheme for energy harvesting is relatively inaccurate for heavily cloudy days. Second, as mentioned earlier, the KMA data are measured at only several points in a city; thus, the past insolation and cloudiness might be a little different in the physical location of our sensor network. These errors can be corrected through data collection at the real position for a long time.

## Figures and Tables

**Table 1:** Notation definitions.

**Symbol**	**Definition**
*α*	The proportional factor for the energy ratio
*A*(*h*)	The total amount of harvested energy for the next *h* hours
*B^p^*(*h*)	The energy that the periodic tasks can use for the next *h* hours
*C^max^*(*h*)	The maximum energy that the aperiodic tasks may use for the next *h* hours
*D*(*h*)	The energy that the periodic tasks will use if they keep the current periods for the next *h* hours
*h^r^*	The remaining time until the solar energy harvesting type is expected to get out of the deficit type

**Table 2:** Simulation parameters.

**Parameter**	**Value**
*Battery range* (*B^min^*, *B^max^*)	0, 10 MJ
*The number of simulation*	30
*The duration of simulation*	120 h
*The rate of the periodic tasks (R1:R2:R3)*	1:1.5:2
*The energy per the periodic packet* (*E)*	28 kJ
*The deadline time of the aperiodic packet*	5 min
*T_s_*, *T_f_*	19, 7 h

**Table 3:** The number of packets missing the deadline. ACSE, adaptive control of packet transmission period with solar energy harvesting prediction.

λ	**Greedy**	**Lazy**	**ACSE**	**Optimal**
4	14	0	3	9
8	37	0	7	29
12	136	11	23	106
